# Evaluation of In Vitro Antimalarial Activity of Different Extracts of *Eremostachys azerbaijanica* Rech.f.

**Published:** 2016

**Authors:** Solmaz Asnaashari, Fariba Heshmati Afshar, Sedigheh Bamdad Moghadam, Abbas Delazar

**Affiliations:** a *Drug Applied Research Centre, Tabriz University of Medical Sciences, Tabriz, Iran. *; b *Department of Pharmacognosy, Faculty of Pharmacy, Tabriz University of Medical Sciences, Tabriz, Iran*

**Keywords:** Antimalaria, *Eremostachys azerbaijanica*, Cell free assay, GC-MS

## Abstract

Six extracts with different polarity from aerial parts and rhizomes of *Eremostachys azerbaijanica* Rech.f., were screened for their antimalarial properties by *cell free *𝛽-hematin formation assay. Dichloromethane (DCM) extracts of both parts of plant showed significant antimalarial activities with IC_50_ values of 0.949 ± 0.061 mg/mL in aerial parts and 0.382 ± 0.011 mg/mL in rhizomes. Bioactivity-guided fractionation of the most potent part (DCM extract of rhizomes) by vacuum liquid chromatography (VLC) afforded seven fractions. Two fractions [100% Ethyl acetate (EtOAC) and 100% Methatol (MeOH)] showed considerable antimalarial activity with IC_50_ values of 0.335 ± 0.033 mg/mL and 0.403 ± 0.037 mg/mL, respectively.

According to GC-MS analysis, the sesquiterpene, steroid and coumarin derivatives are the main constituents of the most potent fractions; therefore, it seems that the anti malarial activity of these fractions may be related to the presence of these types of compounds.

## Introduction

Malaria is one of the most important infectious diseases in the world that caused by an intracellular parasitic protozoa of the genus *Plasmodium* and it is transmitted via the bite of an infected female Anopheles sp mosquito (1, 2). This global disease is still a major cause of severe diseases such as sepsis, responsible for about 655,000 deaths each year and a further 216 million new cases of malaria diagnosed annually (3, 4). It is estimated that more than half of the world᾽s population live in the endemic regions of malaria. Malaria is one of the most important parasitic diseases in southeastern areas of Iran. Sistan va Baluchestan province, which reports about 60% of all the country’s malaria cases, is in the neighborhood of Pakistan and Afghanistan and have considerable cross-border population movement (5, 6).

The first effective treatment (17th century) against the *P. falciparum* parasite was the bark of cinchona tree, which contains quinine, a quinoline alkaloid and by the 19^th ^century, it was still the only known antimalarial agent. Chloroquine was synthesized in 1934 and designated as the choice drug for treatment of malaria in 1946 and is known as the cheapest and commonly used drug for malaria. In recent years, the treatment of malaria has become ineffective because of the increasingly drug resistant by parasite (7, 8 and 9). Nowadays, medicinal plants as an invariably resource are used in the prevention and treatment of malaria in various parts of the world (8, 10).

The discovery of artemisinin from *Artemisia annua* by Chinese scientists in 1960 have provided a new class of highly effective antimalarial drugs and the global demand for artemisinin-based combination therapy (ACT) that has developed since its recommendation by the World Health Organization in 2002 (11, 12). According to the WHO malaria report, in 2013, the use of oral artemisinin-based monotherapies threatens the long-term usefulness of ACTs by developing the emergence or spread of resistance to artemisinin (13). Consequently, the risk of drug resistance and the use of medicinal plants in malaria prevention and treatment lead to the search for new antimalarial compounds from natural origin.

As a continuation of our studies on Iranian plants (14, 15, 16), we have now screened anti malarial properties of different extracts of *Eremostachys azerbaijanica*.

This plant is one of the 60 species of the genus *Eremostachys* (family: Lamiaceae alt. Labiatae; subfamily: Lamioideae) that occur mainly in central Asian countries. Some *Eremostachys* species such as *E. glabra*, *E. laciniata*, *E. lanata* and *E. labiosa* are well distributed in Iran (17, 18). The rhizomes of species belonging to the *Eremostachys* genus are used as local analgesic and anti-inflammatory inducer. Also, this genus has showed antinociceptive, antidepressant, and antibacterial activities (18-23). Previous phytochemical studies on a few species of genus *Eremostachys* revealed the presence of flavonoids such as chrysoeriol glycosides, monoterpene glycosides, iridoid glucosides and ferulic acid derivatives (18, 19). The objectives of this study were to evaluate the antimalarial activity of different extracts from aerial parts and rhizomes of *E. azerbaijanica* and detection of the most potent fractions by GC-MS.

**Table1:** The 50% and 90% inhibition concentrations (mg/mL) of different extracts and VLC fractions from DCM extract of *E. azerbaijanica* rhizomes in cell free 𝛽-hematin formation assay

**Extract or Fraction**	**Yield (%)**	**IC**_50_ **(mg/ml)**	**IC**_90_ **(mg/ml)**
*E. azerbaijanica* aerial parts			
n-Hexan extract	0.76	L	L
DCM extract	0.47	0.949 ±0.061	1.836 ±0.071
MeOH extract	24.68	*	*
*E. azerbaijanica* rhizomes			
n-Hexan extract	0.61	*	*
DCM extract	0.43	0.382 ±0.011	0.800 ± 0.019
MeOH extract	25.91	*	*
VLC fractions from DCM extract of rhizomes			
Fr.10%	1.15	0.590 ±0.013	0.786 ± 0.058
Fr.20%	0.51	0.516 ±0.009	0.768 ± 0.032
Fr.40%	1.34	0.979 ± 0.089	4.376 ± 0.526
Fr.60%	2.63	0.874 ±0.092	1.525 ± 0.002
Fr.80%	4.93	2.135 ±0.005	3.585 ± 0.208
Fr.100%	7.51	0.335 ± 0.033	0.410 ± 0.130
Fr.MeOH 100%	60.32	0.403 ±0.037	0.949 ±0.035
Chloroquine	positive control	0.014 ± 0.003	0.163 ± 0.004

Experiment was performed in triplicate and expressed as Mean ± SD.

**Table 2:** Composition of VLC fractions from DCM extract of *E. azerbaijanica* rhizomes

**Fractions**	**Total identified content (%)**	**Compounds (content %)**
Fr.10%	95.79	Steroides and their derivatives (52.80%), Alkanes (21.46%), Sesquiterpene derivatives (9.49%), Linear ketones (8.21%), Linear alcohols (1.95%), Coumarines (1.88%)
Fr.20%	99.9	Coumarines (63.1%), Fatty acid derivatives (36.8%)
Fr.40%	94.41	Polycyclic aromatic hydrocarbons (66.09%), Steroides and their derivatives (11.40%), Phenolic aldehyde derivatives (7.31%), Linear alcohols (6.08%), Cyclic alcohols (3.53%)
Fr.60%	95.35	Linear alcohol (34.42%), Linear ketones (26.86%), Sesquiterpene derivatives (14.12%), Linear ether (9.38%), Poly cyclic aromatic hydrocarbons (6.52%), Phenolic aldehyde (4.05%)
Fr.80%	99.98	Steroides and their derivatives (49.42%), Linear ketons (24.76%), Linear ether alcohol (8%), Linear ethers (7.74%), Linear alcohol (5.83%), Cyclic alcohol (4.23%)
Fr.100%	100	Sesquiterpene derivatives
Fr.MeOH 100%	99.90	Sesquiterpene derivatives (80.60%), Poly cyclic aromatic hydrocarbons (19.30%)

## Experimental

*Materials*

All the solvents used for extraction and fractionation were from Caledon( Canada), Hematin porcine, Chloroquine diphosphate, Sodium dodecyl sulfate (SDS), Sodium acetate, Magnesium sulfate, Sodium hydrogen phosphate, Sodium chloride, Potassium chloride, Sodium hydroxide, Glucose, and Sodium bicarbonate were purchased from Sigma-Aldrich, Chemical Company (United kingdom), Oleic acid from Fluka (India), Dimethylsulfoxide (DMSO), Hydrochloric acid (HCl), and silica gel 60 (0.040–0.063 mm) from Merck (Germany).

*Plant material:*

The aerial parts and rhizomes of *E. azerbaijanica* Rech.f. were collected respectively during July and September 2012 from Sahand mountains in East Azarbaijan province in Iran 37.759 (37° 45ʹ 32.4″ N) latitude 45.9783 (45° 58ʹ 41.9″ E) longitude and altitude 1850 m above sea level. A voucher specimen (No.738) has been retained in the herbarium of the Faculty of Pharmacy, Tabriz University of Medical Sciences, Tabriz, Iran.

*Extraction*

Air-dried and ground aerial parts and rhizomes of *E. azerbaijanica* (100 g each) were Soxhlet-extracted successively with n-hexane, DCM and MeOH (1.1 L each). All these extracts were separately concentrated using a rotary evaporator at a maximum temperature of 45 °C (24)

*Fractionation*

1.7 g of DCM extract obtained from rhizomes of *E. azerbaijanica* were fractionated by VLC method over silica gel (20 g) with solvent mixtures of increasing polarities: EtOAC/n-Hexan (10: 90), EtOAC/n-Hexan (20 : 80), EtOAC/n-Hexan (40 : 60), EtOAC/n-Hexan (60 : 40), EtOAC/n-Hexan (80 : 20), EtOAC/n-Hexan (100 : 0), and methanol (100). All the fractions were fully dried using a rotary evaporator at a maximum temperature of 45 °C.

*Cell free* *𝛽* *-Hematin Formation Assay.*

Antimalarial activity of plant extracts was studied by the method described by Afshar *et al.* (14, 24) with some modifications. Initially, different concentrations (0–2mg/mL in DMSO) of the extracts and fractions were produced and then the samples were incubated with 3 mM of hematin, 10 mM oleic acid, and 1 M HCl. The final volume was adjusted to 1mL using sodium acetate buffer, pH 5, overnight at 37 °C with constant gentle shaking. Chloroquine diphosphate was used as a positive control.

After incubation, samples were centrifuged (14,000 rpm, 10 min, at 21 °C) and the hemozoin pellet was repeatedly washed with incubation (15min at 37 °C with regular shaking) in 2.5% (w/v) SDS in phosphate buffered saline followed by a final wash in 0.1 M sodium bicarbonate until the supernatant was clear (usually 3–8 washes). After the final wash, the supernatant was removed and the pellets were dissolved in 1mL of 0.1M NaOH before determining the hemozoin content by measuring the absorbance at 400 nm (Spectronic Genesys spectrophotometer). The results were recorded as % inhibition (I%) of heme crystallization compared to negative control (DMSO) using the following equation:

𝐼% = [(AN−AS)/AN] ×100, where AN is absorbance of negative control and AS is absorbance of test samples.

*GC-MS Analysis of potent Fractions*

GC–MS analyses were carried out on a Shimadzu QP-5050A GC–MS system equipped with a DB-1 fused silica column (60 m × 0.25 mm i.d., film thickness 0.25 µM); oven temperature, rising from 50 °C to 310 °C at a rate of 3 °C/min; injector temperature, 280 °C; carrier gas, helium at a flow rate of 1.3 mL/min ; split ratio, 1:10; ionization energy, 70 eV; scan time, 1 s; mass range, 30–600 amu.

*Identification of Components*

Identification of the constituents was based on direct comparison of the retention times and mass spectral data with those for standard compounds, and computer matching with the NIST 21, NIST 107 and WILEY229 library, as well as by comparison of the fragmentation patterns of the mass spectra with those reported in the literature (Adams 2004).

*Statistical Analysis*

All experiments were done in triplicate measurements and presented as the Mean ± SD. Data were analyzed by Excel 2010 Microsoft. The IC_50_ and IC_90_ values were calculated from nonlinear regression analysis.

## Results and Discussion

The *Plasmodium* parasite uses host erythrocyte hemoglobin as a major nutrient source of amino acids. Degradation of hemoglobin produces brown heme crystals which are toxic for parasite (25, 26). To protect itself, the malaria parasite detoxifies free heme via several detoxifying pathways. The prominent way for removing of heme is indicated as crystallization of heme into hemozoin which is a water-insoluble malarial pigment produced in the acidic digestive vacuoles. Therefore, the inhibition of hemozoin or 𝛽-hematin (the synthetic analogue of hemozoin) formation is an important target for studying about antimalarial drugs such as chloroquine and artemisinin and also drug screening programs (25 -29).

**Figure 1:**
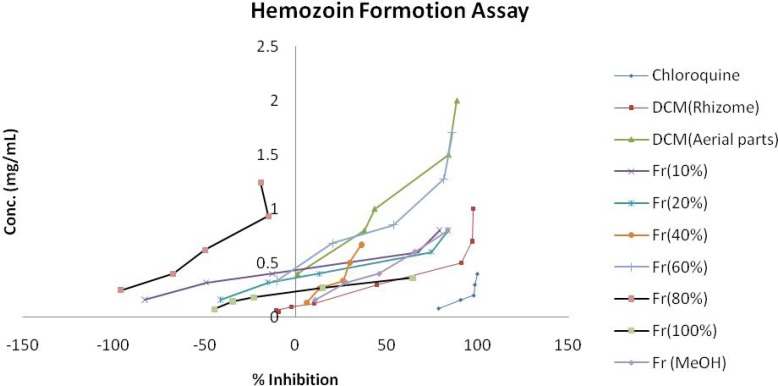
Comparison of % inhibition of heme crystallization between active fractions of *E.azerbaijanica* and chloroquine in cell free 𝛽-hematin formation assay.

**Figure 2:**
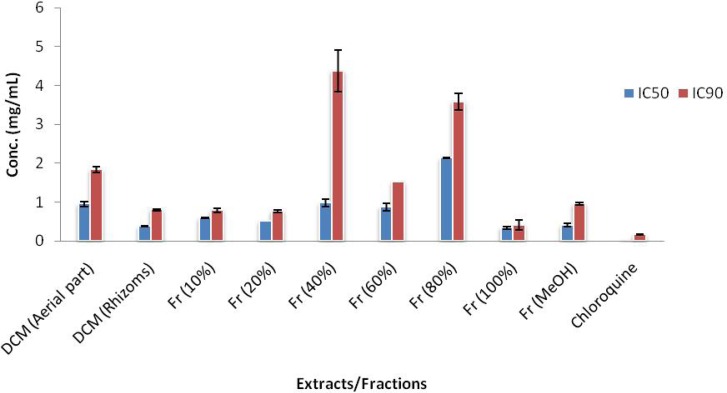
Comparison of IC_50_ and IC_90_ values (mg/mL) of active extracts and fractions of *E. azerbaijanica* and chloroquine solution in cell free β-hematin formation assay. The values were reported as Mean ± SD.

In this study, six extracts of aerial parts and rhizomes and also seven fractions of DCM extract of rhizomes were evaluated for their antimalarial activity by cell free 𝛽-hematin formation assay developed by Afshar *et al.* (15).

Table 1 shows a summary of the cell free 𝛽-hematin formation assay for six different extracts from aerial parts and rhizomes of *E. azerbaijanica* and VLC fractions of DCM extract from rhizomes. According to Table 1. and Figure 1., among six different extracts, DCM extracts of both parts, especially DCM extract of rhizomes, showed the most potent antimalarial activity compared to the standard anti-malarial compound, chloroquine (IC_50_ = 0.014 ± 0.003 mg/mL, IC_90_ = 0.163 ± 0.004 mg/mL) but obtained results revealed that the n-hexane and the MeOH extracts of both parts of *E. azerbaijanica* did not show any significant antimalarial activities. The inhibition of 𝛽-hematin formation expressed as percentage (I%) and standard deviations (𝑛 = 3) are given for each fraction. IC_50_ and IC_90_ values were measured graphically by plotting concentrations versus percentage of inhibition.

IC_50_ values of DCM extracts from aerial parts and rhizomes were 0.949 ± 0.061 and 0.382 ± 0.011 mg/mL, and were 1.836 ± 0.071 and 0.800 ± 0.019mg/mL for IC_90_ values of these extracts, respectively.

*Experiment was performed in triplicate and expressed as Mean ± SD.*

After fractionation of the most potent extract (DCM extract of rhizomes) by VLC method over silica gel, the results showed that among the seven different polarity fractions, 100% EtOAC and 100% MeOH fractions had considerable anti-malarial activity with IC_50_ values of 0.335 ± 0.033 and 0.403 ± 0.037 mg/mL and IC_90_ values of 0.410 ± 0.130 and 0.949 ± 0.035 mg/mL, respectively. Two other fractions of VLC (10% EtOAC/n-Hexane, 20% EtOAC/n-Hexane fractions) demonstrated remarkable anti-malarial effects with close IC_50_ and IC_90_ values (Table 1. and Figure 2.).

GC-MS analysis of the most potent fractions showed various compounds with different functional groups. The results of GC-MS analysis of seven VLC fractions from DCM extract of rhizomes have given in Table 2.

The main groups of compound identified by GC-MS analysis of fractions were steroide derivatives, sesquiterpene derivatives, coumarins, fatty acid derivatives, polycyclic aromatic hydrocarbons and some linear structures with functional groups such as aldehyde, ketone, ether and alcohol.

Studying the previous literatures showed that terpenes, steroids, coumarines, flavonoides, phenolic acids, lignanes, xanthones and anthraquinones exhibited antiplasmodial activity in different antimalarial assays (30-32). In 100% EtOAC and 100% MeOH fractions, sesquiterpene derivatives were identified as the major active constituents and could explained the potent activity of these fractions. In the case of 10% EtOAC/n-Hexane and 20% EtOAC/n-Hexane fractions, the potent antimalarial activity might be due to the high content of sesquiterpenoid lactones and coumarin derivatives. In addition, the IC_50_ and IC_90_ values could be reduced in 20% EtOAC/n-Hexane fraction by entirely removing the lipids and more purification of potent constituents. These results are in agreement with previous research that showed the synergistic effect of lipids and other fatty acids in the mixtures of extracts with oleic acid in the assay (14). 

However, according to the Table 1. and Figure 2. single fractions of VLC were not potent enough, comparing to total DCM extract of rhizomes that can be due to the synergistic or combination effect of seven fractions compounds of VLC. 

## Conclusion

From the 6 extracts with different polarity of aerial parts and rhizomes of *E. azerbaijanica*, the DCM extract of both parts were the most active extracts in cell free 𝛽-hematin formation assay and the preliminary phytochemical study on the VLC fractions of the most potent part (DCM extract of rhizomes) persuade us to focus on purifying the active components of these extracts and investigating further on animal models for *in vivo* evaluation.
